# Marine Natural Products and Coronary Artery Disease

**DOI:** 10.3389/fcvm.2021.739932

**Published:** 2021-09-21

**Authors:** Bo Liang, Xin-Yi Cai, Ning Gu

**Affiliations:** ^1^Nanjing University of Chinese Medicine, Nanjing, China; ^2^Nanjing Hospital of Chinese Medicine Affiliated to Nanjing University of Chinese Medicine, Nanjing, China

**Keywords:** marine natural products, coronary artery disease, review, marine, cardiovascular disease

## Abstract

Coronary artery disease is the major cause of mortality worldwide, especially in low- and middle-income earners. To not only reduce angina symptoms and exercise-induced ischemia but also prevent cardiovascular events, pharmacological intervention strategies, including antiplatelet drugs, anticoagulant drugs, statins, and other lipid-lowering drugs, and renin–angiotensin–aldosterone system blockers, are conducted. However, the existing drugs for coronary artery disease are incomprehensive and have some adverse reactions. Thus, it is necessary to look for new drug research and development. Marine natural products have been considered a valuable source for drug discovery because of their chemical diversity and biological activities. The experiments and investigations indicated that several marine natural products, such as organic small molecules, polysaccharides, proteins, and bioactive peptides, and lipids were effective for treating coronary artery disease. Here, we particularly discussed the functions and mechanisms of active substances in coronary artery disease, including antiplatelet, anticoagulant, lipid-lowering, anti-inflammatory, and antioxidant activities.

## Introduction

Coronary artery disease (CAD) mostly results from atherosclerosis, which may cause arteries to be narrowed or clogged by cholesterol and fat deposits (1). Furthermore, atherosclerosis can result in myocardial ischemia and hypoxia. CAD can be divided into acute coronary syndromes and chronic coronary syndromes (1). Acute coronary syndromes attack when a portion of the heart is completely cut off by total blockage of a coronary. This is usually due to a sudden closure from a blood clot forming on top of a previous narrowing. Chronic coronary syndrome always accompanies chest pain because of the lack of oxygenated blood supply. It was demonstrated that CAD had been considered the primary hazard to adults in all age groups in the US. In addition, it could lead to a destructive influence on younger generations and their relatives (2). CAD is also referred to as the most popular angiocardiopathy and a significant risk regarding public hygiene (3). Another investigation also indicated that CAD was the major reason for mortality in the world, especially in low- and middle-income earners (4). In conclusion, CAD is a harmful disease that is worthy of more attention.

CAD has several risk factors, including family history, age, obesity, smoking, passive smoking, physical activity, dyslipidemia, diabetes, hypertension, H-type hypertension, sleep, and so on (3). Hence, to not only reduce angina symptoms and exercise-induced ischemia but also prevent cardiovascular events, pharmacological intervention strategies, including antiplatelet drugs, anticoagulant drugs, statins and other lipid-lowering drugs, and renin–angiotensin–aldosterone (ACE) system blockers, are conducted. It was reported that the excitation and gathering of thrombocytes could induce arterial coronaria thrombogenesis. As a consequence, to achieve a balance between ischemia and hemorrhage, we often used antiplatelet drugs such as aspirin and P2Y12 depressors to treat myocardial infarction and percutaneous coronary intervention postoperatively. Furthermore, it was believed that CAD patients must receive statin therapy regardless of low-density lipoprotein cholesterol (LDL-C) standards to lower the risk of angiography accidents. In regard to ACE suppressants, they could decrease the incidence rate of vascular sickness in left ventricular dysfunction patients (5).

**Figure 1:**
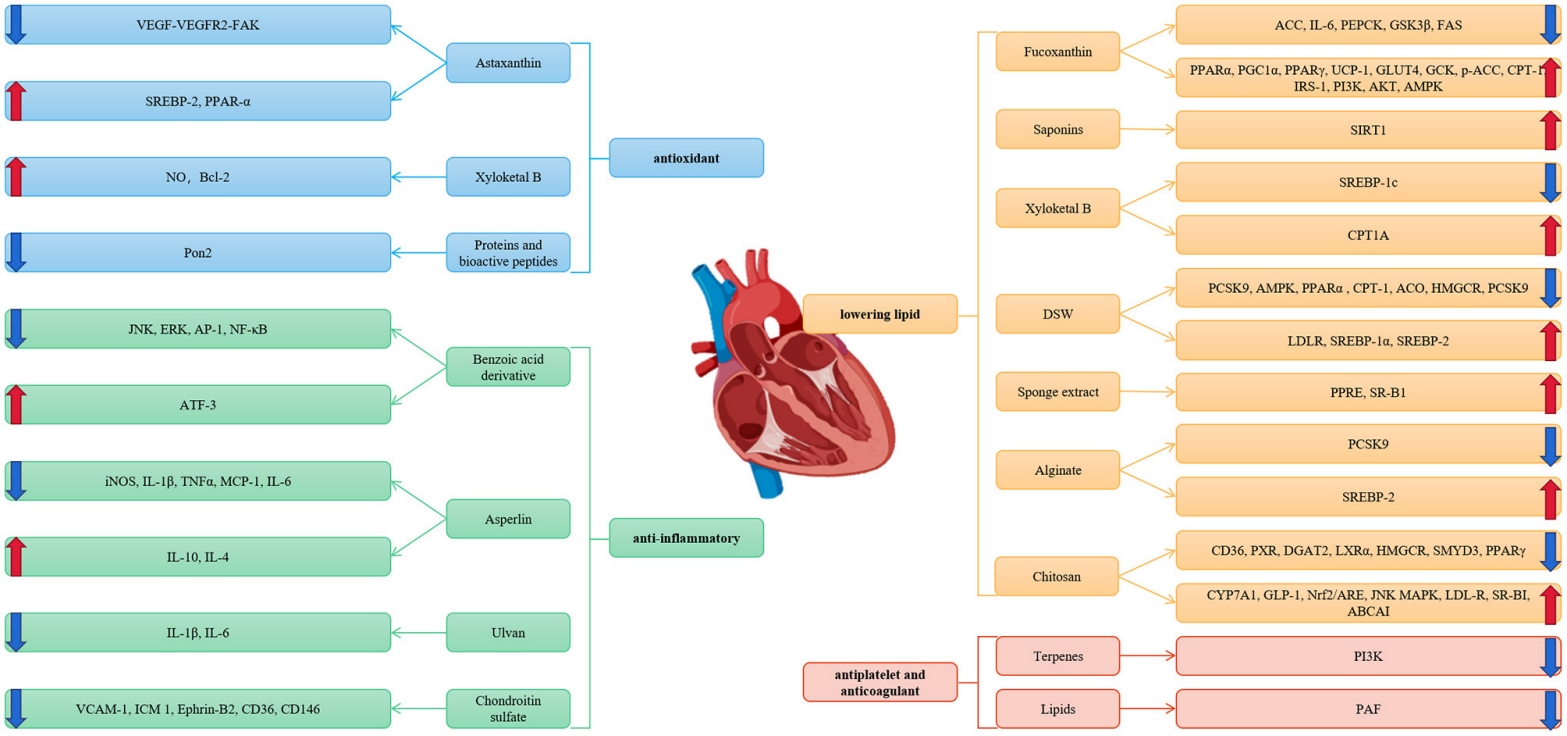
Marine natural products and coronary artery disease.

However, the existing drugs to treat CAD are incomprehensive and have some adverse reactions. Thus, it is necessary to look for new drug research and development. Marine natural products have been considered a valuable source for drug discovery because of their chemical diversity and biological activities. Several studies have proven that a majority of marine natural products have the bioactivity mentioned above. Therefore, marine natural products have been proven to have a function in the treatment of CAD. In this passage, we reviewed the up-to-date studies of several marine active materials, such as organic small molecules, polysaccharides, proteins, bioactive peptides, and lipids, related to CAD and further probed the functions and mechanisms of these marine active materials on CAD (Figure 1).

## Organic Small Molecules

### Fucoxanthin

Fucoxanthin is a carotenoid containing oxygen that is extracted from brown algae (6). Recent studies have suggested that fucoxanthin prevented lipids from overoxidation and restrained the accumulation of lipids. In addition, fucoxanthin downregulated transcription factors related to adipogenesis, such as sterol regulatory element-binding protein 1c and peroxisome proliferator-activated receptor. Meanwhile, fucoxanthin not only decreased the expression of fatty acid synthase but also increased adipose triglyceride lipase and the phosphorylation of the production of lipase sensitivity to the hormone that is used for lipolysis (7). Studies have also demonstrated that fucoxanthin promotes peroxisome proliferator-activated receptor (PPAR) α, primordial germ cell (PGC) 1α, PPARγ, and uncoupling protein (UCP)-1 so that the expenditure of energy, β-oxidation, and lipogenesis can be increased. Furthermore, it suppressed the gene expression of acety1-CoA carboxylase (ACC) and interleukin (IL)-6. As a result, the accumulation of adipose tissue, insulin resistance, cholesterol thickness, and triglyceride concentration according to serum and inflammation could be significantly improved (8). In regard to UCP1 (9) and glucose transporter 4 (GLUT4), fucoxanthin upregulated them to consume energy by fatty acid oxidation and the production of heat (10). Additionally, fucoxanthin remarkably reduced the blood glucose level by increasing the expression of glucokinase (GCK) mRNA and restrained the expression of phosphoenolpyruvate carboxykinase mRNA. In skeletal muscle, fucoxanthin elevated the glycogen content and GLUT4 and GSY protein expression as well as suppressed glycogen synthase kinase (GSK)3β protein expression. Consequently, glycogen synthesis could be promoted. Finally, fucoxanthin promoted the protein expression of PPARα, p-ACC, and carnitine palmitoyl-transferase (CPT)-1 and restrained FAS protein expression in the liver to reduce blood lipid levels. Mechanistic investigations demonstrated that insulin receptor substrate (IRS)-1/phosphatidylinositol 3-kinase (PI3K)/kinase B phosphorylation (AKT) and AMP-activated protein kinase (AMPK) signaling protein expression were observably increased with the use of fucoxanthin (11).

### Saponins

Sea cucumber saponins (SCSs) can be defined as secondary metabolites coming from sea cucumbers, and SCSs are a group of glycosides, the aglycones of which are triterpene or spirostane compounds (12). Studies have demonstrated that SCSs could be competent for antiatherosclerosis activity. To explain the mechanism, reasons can be divided into two sides. On the one hand, it might be the function of SCSs to regulate lipid metabolism and glycometabolism. Recent studies have indicated that SCS treatment dose dependently decreases the levels of lipids in serum in rats fed a cholesterol-rich diet. In particular, ApoE^−/−^ mice accelerated the disappearance of plaques by 56.9% with an 8-week treatment of 0.07% SCSs (13). Further research showed that adiponectin, which is released by sea cucumber saponins, is the main functional ingredient to ameliorate the metabolism of lipids and glucose. Studies have suggested that adiponectin promotes sirtuin 1 (SIRT1) expression. Due to the activation of SIRT1, sterol regulatory element-binding protein (SREBP)-1c, stearoyl-CoA desaturase (SCD)-1, and fatty acid synthase (FAS) expression could be restrained not only at the mRNA level but also at the protein level. Studies have indicated that overexpression of SIRT1 elevates the level of PPARα and its coactivator peroxisome PGC-1α, contributing to the promotion of the oxidation of hepatic fatty acids. In addition, SIRT1 could stimulate the mRNA level of GCK in the liver, resulting in the catalysis of glucose phosphorylation, which is the first step in glycolysis (14). In conclusion, SCSs ameliorate lipid metabolism and glycometabolism by releasing adiponectin to promote SIRT1 signaling so that SREBP-1c, FAS, SCD-1, and PGC-1α could be restrained, and PPARα and GCK could be elevated, which are all downstream genes of SIRT1 that suppress lipid synthesis and promote fatty acid β-oxidation and the glycolysis pathway (13). Other scholars have found that *Thelenota ananas* saponin extract holothurin A (desHA) affects the metabolism of cholesterol in foam cells induced by ox-LDL. The reasons can be listed as follows. DesHA suppressed the liver X receptor (LXR)/AKT/AMPK pathway to adjust 3-hydroxy-3-methylglutaryl (HMG)-CoA reductase and NOS. As a result, desHA significantly restrained endocellular cholesterol synthesis and elevated intracellular cholesterol outflow. Furthermore, studies have indicated that SCSs decrease triglyceride (TG), total cholesterol (TC), and LDL-C levels and elevate high-density lipoprotein cholesterol (HDL-C) levels in the serum of mice and rats. On the other hand, SCSs obtained anti-inflammatory activity by suppressing tumor necrosis factor-α (TNF-α) and lowering the expression of TNF-α in the aorta of ApoE^−/−^ mice. In addition, SCSs restrained IL-1β, IL-6, and MCP-1, which could be defined as inflammatory factors (12). Finally, SCSs in the diet might inhibit proinflammatory cytokine expression in vascular and peritoneal macrophages (13).

### Astaxanthin

Astaxanthin is a natural compound with bioactivity, which is classified as xanthophyll, existing in microalgae and marine natural products. Notably, astaxanthin possessed strong antioxidant activities because of its characteristic molecular structure, which could result in the quenching of oxygen in the singlet state and the elimination of free radicals. An increasing number of studies have demonstrated that astaxanthin has the ability to ameliorate oxidative stress, reduce inflammation, and decrease lipids and glucose.

Studies have indicated that astaxanthin could suppress the oxidation of LDL and promote HDL cholesterol and adiponectin levels (15). Another investigation suggested that taking CDX-085 as the new-type astaxanthin prodrug orally could indicate its distribution in lipoproteins. It is worth noting that the levels of total cholesterol, aortic arch atherosclerosis in LDLR^−/−^ mice, and triglycerides in ApoE^−/−^ mice were restrained by CDX-085 (16). Further studies showed that the replenishment of astaxanthin in a short period and long period stimulated reverse cholesterol transport (RCT) in C57BL/6J and ApoE^−/−^ mice, respectively. RCT is known to have major atheroprotective activity against HDL. In addition, astaxanthin might lighten not only the plaque proportion of the aortic sinus but also cholesterol in the aorta in mice (17). Other studies indicated that astaxanthin-fed rats had lower cholesterol levels of LDL-C and higher levels of HDL-C. It was also reported that astaxanthin suppressed the level of thiobarbituric acid reactive substances to express oxidation resistance (18). Certain investigations inferred that if we supple astaxanthin, the regulatory cholesterol pool might be inhibited, and then, the SREBP-2 pathway might be promoted so that LDLR could be expressed at higher levels. It was also speculated that astaxanthin promoted PPARα based on the experimental phenomenon that CPT-1, acyl-CoA oxidase, and UCP-2 were activated and were PPARα target genes. PPARα participates in the process of preventing cellular oxidative injury under normal cellular metabolism or oxidative stress conditions (19). Further studies showed that astaxanthin restrained fat degeneration in the liver in mice by differentially regulating PPARα and PPARγ, suppressing Akt, and elevating the activation of hepatic autophagy (20).

Some findings suggested that astaxanthin prevented the VEGFR2-p-Tyr397-focal adhesion kinase (FAK) signaling axis from being activated, which was induced by homocysteine (Hcy), to suppress endotheliocyte dysfunction. If Hcy levels in plasma increase, blood vessel endothelial cells are seriously destroyed (21). Another study indicated that astaxanthin restrained mitochondrial dysfunction and oxidative damage to block the cardiotoxicity induced by Hcy both *in vivo* and *in vitro* (22). Further studies investigated whether astaxanthin suppressed the activation of macrophages (23) and promoted the ability of neutrophil granulocytes to phagocytose and sterilize. As a consequence, both lipids and proteins sustained less oxidative damage due to astaxanthin (24).

### Xyloketal B

Xyloketal B is a new-style compound that has an extraordinary chemical structure and is extracted from *Xylaria* sp. (12). Previous studies have shown that xyloketal B protects against oxidative injury in endotheliocytes induced by oxLDL by suppressing NADPH oxidase-derived ROS generation, facilitating the production of NO, and recovering the expression of Bcl-2 (25). Another study showed that in high-fat diet-fed ApoE^−/−^ mice, xyloketal B reduced the atherosclerotic plaque area not only in the aortic sinus but also all over the aorta by relying on the dose. Moreover, oxidative endothelial dysfunction as well as the reduction in the bioavailability of NO are principal for CAD development. Recent studies have indicated that xyloketal B is effective in suppressing vascular oxidative stress levels and improving the integrity of the injured endothelium as well as the vasorelaxation of NO-dependent aortas in atherosclerotic mice. In addition, xyloketal B outstandingly transformed both eNOS and Akt phosphorylation levels but did not change total eNOS and Akt expression in cultured human umbilical vein endothelial cells (26). Several findings demonstrated that xyloketal B reduced lipids through the lipid-regulated activation of the SREBP-1c pathway. Xyloketal B promoted CPT1A expression and suppressed SREBP-1c expression, and the downstream targeting enzymes of SREBP-1c, such as ACC1, ACL, and FAS, were also inhibited. Xyloketal B was reported to decrease the accumulation of lipids in HepG2 cells treated with FFAs (27).

### DSW

Recent studies demonstrated that DSW could suppress the increase in cellular cholesterol levels induced by high glucose or FFA/glucose by promoting the transcription of LDLR and ApoA1 and restraining the expression of PCSK9 mRNA in HepG2 hepatic cells. Furthermore, it is key to determine whether 3-hydroxy-3-methylglutatryl-CoA reductase (HMGCR) expression and/or AMPK phosphorylation take part in the hypocholesterolemic functions of DSW and the proportion of Mg in DSW (28). The findings also indicated that DSW markedly suppressed the activity of intracellular triglycerides and glycerol-3-phosphate dehydrogenase in 3T3-L1 adipocytes. DSW also restrained adipocyte differentiation, lipogenesis, and adipocytokine gene levels as well as elevated lipolysis and fatty acid oxidation gene levels (29). Additionally, DSW was reported to decrease serum lipids by inhibiting the levels of TG and TC in serum and suppressing AMPK, PPARα, CPT-1, and ACO expression (6). Another investigation also indicated that DSW inhibited HMGCR and PCSK9 expression, promoted the phosphorylation of AMPK, and elevated LDLR, SREBP-1α, and SREBP-2 expression (6).

### Terpenes

Terpenes are isolated from marine products and have been demonstrated to have anti-inflammatory, antimicrobial, and antiangiogenic functions (30). Previous studies indicated that dichotomanol could suppress the gathering of platelet-rich plasma induced by adenosine diphosphate or collagen, but pachydictyol A and isopachydictyol A could not. Meanwhile, dichotomanol was incapable of restraining washed platelets. Nevertheless, pachydictyol A and isopachydictyol A could suppress the accumulation of collagen- or thrombin-induced WP. The diterpenes mentioned above could restrain coagulation and thrombin catalysis (31). According to recent molecular mechanism research, compared with pachydictyol A and isopachydictyol A, the lowest electronic density of dichotomanol tended to have a better activity to suppress the catalytic activity of thrombin (32). Recent investigations indicated that frondoside A, which is a marine-derived triterpenoid, could suppress the PI3K pathway in platelets to inhibit the formation of thrombi (33).

### Benzoic Acid Derivatives

It was proven that one novel anthranilic acid derivative, which was extracted from a Philippine sponge, could inhibit proinflammatory cytokines by restraining JNK, ERK, activator protein-1, and NF-κB as well as promoting the ATF-3 signaling pathway (34).

Another investigation found that R-/S-HPABA, which was extracted from marine natural products, had an anti-inflammatory function. An *in vitro* experiment indicated that R-/S-HPABA strongly suppressed more aggregation of platelets, which was induced by ADP, collagen, and arachidonic acid, in rabbit plasma enriched with platelets than in the control group. In regard to the extent that the aggregation of platelets was inhibited, we could conclude that it was similar to that of aspirin. Significantly, R-/S-HPABA inhibited the thromboxane B2 level and elevated F1α generation. In addition, R-/S-HPABA could lower the weight of carotid thrombosis (35).

### Sponge Extract

SR-B1 is one type of HDL receptor significant for AS. HDL could combine SR-B1 with the purpose of regulating the transport of cholesterol so that AS might be regulated (12). Compounds purified from tetracyclic merosesquiterpene, which was extracted from the sponge *Hyrtios digitatus* in Australia, promoted SR-B1 activity in HepG2 cells in a dose-dependent manner, which upregulated the activity of SR-B1 in HepG2 cells (36). Investigations have also indicated that sponge extracts might have antiatherosclerotic effects by upregulating PPAR response elements and SR-B1 expression (12).

### Asperlin

Asperlin was extracted from marine natural products and has been demonstrated to have antifungal and anti-inflammatory activities *in vitro*. Recently, we found that asperlin significantly inhibited the formation of foam cells induced by LPS. Meanwhile, it elevated the external flow of cholesterol in RAW264.7 macrophages. It was also reported that if asperlin was added, proinflammatory divisors induced by LPS could be inhibited in RAW264.7 macrophages, iNOS, IL-1β, and TNFα expression might be restrained, and IL-10 and IL-4 expression could be promoted. As a consequence, macrophage polarization had an outstanding transformation. In ApoE^−/−^ mice fed a high-fat diet, it was shown that taking asperlin orally could markedly lower the formation of aortic atherosclerotic plaques *in vivo* by inhibiting the dilatation of the aorta and diminishing the proportion of atherosclerotic lesions (37). Furthermore, asperlin lowered proinflammatory cytokine levels in serum, such as MCP-1, TNF-α, and IL-6, on the basis of unaltered lipid profiles (12).

## Polysaccharides

### Fucoidan

Fucoidan was exacted from brown alga (38). Fucoidan has been demonstrated to possess antioxidant, lipid-lowering, and antiangiogenic activities. In one study, LMWF was indicated to have oxidation resistance. In addition, LMWF can be used in anticoagulant therapy. The findings showed that ApoE^−/−^ mice regulate the immune response by promoting the IL-6, IL-10, p-SAPK/JNK, VEGF, and FGF/FGFR signaling pathways. LMWF has the potential to treat arteriosclerosis (39). Furthermore, LMWF could also exert anti-inflammatory effects by suppressing IL-6 and promoting IL-10. In addition, LMWF elevated aortic CD11b mRNA levels and downregulated intimal aortic CD11b expression, thus, protecting against macrophage translation into foam cells and smooth muscle cell transfer to the aortic intimal layer. As a consequence, atherosclerotic plaques could be suppressed. Another investigation suggested that triglycerides and ox-LDL could be lowered by LMWF so that arteriosclerosis injury could be restrained (40).

Recent findings showed that fucoidan could decrease lipids in C57BL/6J mice fed a high-fat diet. The mechanism studied by concentrating on the liver as well as the small intestine is listed as follows. Fucoidan could promote the signaling pathways of PPARα, LXRβ/ABC, SR-B1, LDLR, CYP7A1, NPC1L1, ABCG5, and ABCG8 as well as suppress PCSK9 and SREBP expression (41).

Another investigation indicated that *in vivo* fucoidan might protect against the formation of microvascular thrombi resulting from its anticoagulative activation (42).

Further studies indicated that fucoidan could restrain angiogenesis by suppressing signaling pathways such as the TGF, HIF-1/VEGF, miR-29b-DNMT3B-MTSS1-mediated TGF signaling, HIF-1/VEGF-C, PI3K/Akt/mTOR, and JNK/c-Jun/AP-1 pathways. It was also indicated that fucoidan had two-way regulation of angiogenesis, of which the significant structure divisor was 20- to 30-kDa Mw (43).

### Alginate

Alginate (ALG) is mostly collected from brown seaweed (12). Previous studies reported that a 2% Ca-ALG diet inhibited cholesterol in plasma in rats fed high-cholesterol food (44). After 4 weeks of ALG gavage treatment, the body weight and the accumulation of lipids, TGs, and TC were suppressed in high-fat diet-fed mice. Additionally, ALG could ameliorate the standard of serum glucose as well as serum lipopolysaccharide. Furthermore, several RNA genes were inferred that were related to the metabolism of both lipids and carbon compounds (45). AOS was reported to inhibit LDL-C in plasma by adjusting the LDLR. Specifically, it was mostly related to the promotion of SREBP-2 and the suppression of PCSK9 (46). Recent studies found that amidated ALG obtained greater lipid-lowering ability, and thus, hydrophobic amidated ALG possessed a stronger ability to lower lipids than hydrophilic ALG (47).

### Ulvan

Ulvan is a sulfated heteropolysaccharide that is extracted from green algae (38). Many findings have indicated that ulvan has a function in atherosclerosis. Some constituents of ulvan were proven to have inflammation resistance with high MW and could suppress IL-1β and IL-6 levels to some degree (48). Recent investigations showed that the higher the degree of sulfation of ulvan is, the higher its coagulant resistance (49). It was also investigated whether ulvan possessed outstanding oxidant and hyperlipidemic resistance. During one study, purified ulvan3, whose percentage compositions of glucuronic acid and sulfate radicals were the largest, while its average molecular weight (MW) was the lowest, was proven to obtain quite stronger oxidation resistance (50). With a 4-week ulvan diet, the levels of TG were restrained in high-fat diet-fed rats (38).

### Carrageenan

Studies have demonstrated that carrageenan can suppress TC, TG, and LDL-C standards in serum and upregulate HDL-C concentrations in high-fat diet-fed mice with a low MW rather than a high MW (51).

### Chitosan

Chitooligosaccharide (COS) is degraded mainly by enzymes from chitosan (52). Research inferred that COS could suppress the TLR4/NF-κB signaling pathway to protect against inflammation resulting from LPS and dextran sulfate sodium in IPEC-J2 cells and mice (53). Another study indicated that COS could restrain TG, LDL, and TC in both the serum and liver to decrease steatosis in the liver. Lipid accumulation could be improved. The mechanism might be related to the inhibition of the mRNA and protein expression of a cluster of differentiation (CD)36, PXR, diacylglycerol acyltransferase-2, LXRα, and PPARγ (52). It was also reported that COS could promote the expression of not only the cholesterol-degrading enzyme cholesterol 7-α-hydroxylase but also incretin GLP-1 and suppress both HMGCR transcription and expression, which might especially restrain cholesterol synthesis. Recent findings demonstrated that COS could downregulate SET and SMYD3 to regulate HMGCR, ultimately lowering lipids. Furthermore, COS could regulate the inordinate metabolism of both glucose and lipids by adjusting enteric microorganisms as well as the signaling pathways induced by SMYD3 (54). Recent investigations indicated that the COS diet could ameliorate both the lipid properties and oxidation resistance of people suffering from coronary heart disease by stimulating probiotic descriptions and quantities in enteric flora (55). Another finding showed that COS could promote the Nrf2/ARE pathway induced by p38 as well as JNK mitogen-activated protein kinases to protect against oxidative stress and cordis apoptosis (56). Additionally, COS might promote LDL-R and SR-BI as well as macrophage ABCAI expression in the liver and eventually result in the downregulation of AS levels and non-HDL levels in the plasma in ApoE^−/−^ mice (57). Chitoheptaose was also proven in an experiment to obtain oxidant, inflammatory, and apoptotic resistance so that it could defend the heart in a model of myocarditis (58). Recent studies indicated that if chitosan sulfate was embedded into the perivascular chamber, remedial angiogenesis could be achieved to restrain the initial indication of atherosclerotic inflammation (59).

### *Enteromorpha prolifera* Polysaccharides

*Enteromorpha prolifera* polysaccharides (EPPs) are derived from green algae (12). Studies have demonstrated that EPPs can promote HIF-1α expression to improve acute coronary syndromes *in vivo* and *in vitro* (60). It was indicated that sulfated EPPs possessed coagulant resistance, and the 206-kDa outcome could efficiently lengthen APTT (61). EPP degradation was proven to have antioxidant effects by suppressing the expression of miR-48, miR-51, and miR-186 and promoting the expression of SKN-1 and DAF-16; thus, the accumulation of endocellular active oxide species and DNA injury could be improved (62). With EPP treatment for 6 weeks, the TG, TC, and LDL in the liver and liver weight were all lowered in male Sprague–Dawley rats. As a consequence, EPPs might have an antihyperlipidemic effect (63).

### Porphyran Polysaccharides

The findings indicated that porphyran polysaccharides (PPs) inhibited the levels of TG and TC as well as the LDL-C/HDL-C ratio in experiments after the 28-day drug delivery route. Another study demonstrated that the addition of PPs could protect against liver injury caused by a high-fat diet (64). It was also reported that PPs could suppress the NO standard so that lipid peroxidation might be prevented (65). Recent studies indicated that PP therapy observably inhibited the levels of triacylglycerol in plasma, TC, LDL cholesterol, and elevated HDL cholesterol in male Sprague–Dawley rats (66). It was proven that PPs possessed oxidation resistance, which was controlled by their MW; thus, the degradation product or ramification of PPs might obtain higher oxidation resistance than PPs themselves (67). Further studies demonstrated that sulfating PPs could lengthen the activated partial thromboplastin time (APTT), but alkali-treated PPs could not. Meanwhile, the level of PP coagulant resistance depends on the extent of sulfation and sulfate group distribution (68). Previous studies showed that PPS at 500 μg/ml could block NF-κB activation, which is a proinflammatory transcription factor, and, thus, entirely suppressed the generation of NO as well as the expression of iNOS in RAW264.7 cells induced by LPS. Another finding also indicated that PPs might restrain proinflammatory factor production and release. Collectively, we could conclude that PPs obtain anti-inflammatory function (67), of which the degree depends on the MW (69).

### Chondroitin Sulfate

It was reported that one type of concentrated chondroitin sulfate had been proven to obtain less anticoagulant ability than heparin but more than enoxaparin through the experiments of the APTT and thrombin time. Another purified protein test also showed that chondroitin sulfate significantly elevated the effect of factor Xa with antithrombin suppressing thrombase and Xa divisors. However, it had nothing to do with the gathering of thrombocytes in high-platelet adtevak (70). Recent studies demonstrated that fucosylated chondroitin sulfate possessed observable fibroblast growth factor 1 and FGF2-binding affinities. FGF1 and FGF2 are growth elements that participate in elevating endothelicyte transference, levicellular propagation, and angiopoiesis (71). One experiment indicated that even if the patterns of sulfation differed, chondroitin sulfate obtained similar antithrombotic activity; thus, the influence of the chondroitin sulfation pattern could be less than the molecular weight with regard to the antithrombosis and anticoagulation activities (72). In regard to recent studies, we confirmed that FCS9-18 oligomers have antithrombotic activity without any adverse reaction because they might react with several blood coagulating proteins, and then, a majority of them could be discharged from the kidneys to protect against thrombosis; meanwhile, their total had nothing to do with hemorrhage, hypotension, and thrombocyte gathering through blood circulation (73).

In certain investigations, it was indicated that if chondroitin sulfate E was provided exogenously, the combination of VEGF-A and receptor-type protein tyrosine phosphatase beta/zeta (RPTPβ/ζ) could be suppressed in human endotheliocytes. Without RPTPβ/ζ, VEGF-A is incapable of inducing endotheliocyte removal (74).

Further studies indicated that chondroitin sulfate (CS) possesses anti-inflammatory action by suppressing white blood cell recruitment and acting as a standard proinflammatory cytokine (75). CS has been demonstrated to protect the heart due to its regulation of endothelial and monocytic proinflammatory activation as well as the formation of xanthoma cells. Specifically, CS inhibited vascular cell adhesion molecule 1, intercellular adhesion molecule 1, and ephrin-B2 expression and elevated inflammatory endotheliocyte transference. In addition, CS restrained the formation of xanthoma cells *in vivo*, the expression of CD36 and CD146, and the intake and accumulation of oxidized low-density lipoprotein in cultured sensitized mononuclear cells as well as macrophagocytes (76). According to acute peritoneal inflammation experiments, CS possesses anti-inflammatory action (77).

## Proteins and Bioactive Peptides

It is well-known that proteins and bioactive peptide metabolites are good at oxidation resistance, hyperpiesia resistance, and other influences in defending the heart (78).

In regard to the hypolipidemic activity of the bioactive peptides through which AS deterioration could be postponed, studies have indicated that porphyra peptide suppresses the increase in rat weight with hyperlipidemia, decreases TC, TG, and LDL-C in the serum, and elevates HDL-C. It was also reported that sea cucumber peptides had a similar function to that mentioned above. Furthermore, protein hydrolysate extracted from salmon was demonstrated to have lipid-lowering activity and inflammation resistance by suppressing factors such as IL-1β, IL-6, TNF-α, granulocyte-macrophage colony-stimulating factor, and granulocyte colony-stimulating factor in the plasma. Studies also showed that collagen peptides isolated from marine cucumber could lower the standard of malondialdehyde, suppress lipid superoxidized production, increase NO synthase activity, and stimulate NO production. As a consequence, vascular endotheliocytes might be defended to protect against atherosclerosis and AS (12). Another investigation suggested fish protein as well as oxidation resistance according to the heart and liver instead of plasma. Studies also found that the Pon2 gene in the liver was suppressed in cod–scallop dietary mice, which indicated a decrease in urban oxidation (78). It was also reported that with undigested goby protein, goby protein hydrolysates or fermented sardinelle (*Sardinella aurita*) protein hydrolysate treatment, high fat and fructose diet dietary rats not only obtained a great decrease in TC, TG, and LDL-C in the serum and TC and TG in the liver but also caused an outstanding increase in HDL-C in the serum. Furthermore, both thrombin and prothrombin, which gave rise to arteriosclerosis and cruor, were suppressed. Pancrelipase activity was also inhibited to suppress the accumulation of lipids (79). Fermented sardinelle protein hydrolysates could greatly protect against heart attack by reinstating arteriosclerotic fingers and defending the heart and aorta organization structure (80).

A recent investigation suggested that one type of protein extracted from *Anemonia viridis* was demonstrated to inhibit endotheliocyte propagation as well as angiogenesis at 14 nM, and the mechanism of its activity could be Kunitz-form suppressant, which might have reciprocity with one integrin due to one arginine glycin aspartate element. Furthermore, this type of arginine glycine aspartate element was greatly revealed in a solvent, the mechanism of which was useful for improving the present antiangiogenic treatment, mainly with regard to the combination of antiangiogenic compounds and VEGF (81). Studies have indicated that cod protein has anti-inflammatory effects induced by sophisticated arginine, glycine, taurine, and lysine (82).

Recent studies suggested that one new type of protease called SK could restrain thrombosis within limits by improving the blood-clotting system depending on the dosage. Under most circumstances, SK functioned better than urokinase. Four important pathways, arachidonic acid, nicotinate, sphingolipid, and nicotinamide, were discovered to demonstrate that the mechanism by which SK influences the formation of arteria carotis communis thrombogenesis is through suppressing the vasoconstriction, gathering, conglutination, and liberation of thrombocytes, correcting endotheliocyte dysfunction and postponing thrombogenesis (83). Another study also found that spumigins from blue–green algae whose construction imitated the D-Phe-Pro-Arg sequence had a significant function on the combination of the active sites of serine proteases thrombin and factor Xa. Studies have indicated that (2S,4S)-4-methylproline central core spumigins might possess great potential as novel immediate thrombin suppressants (84). Recent studies demonstrated that VPH and VPH-I might obtain similar lower blood pressure ability with captopril (~9 mmHg at t = 4 h) (85).

## Lipids

It was reported that lipids extracted from microalgae of *Chlorococcum* sp. have inflammatory resistance and thrombotic resistance. Among the lipid components that could activate platelets as well as antithrombi, glycolipids, and phospholipids possessed the highest activity (86). Another investigation also indicated that salmon polar lipids (PLs) could exert antithrombotic effects by suppressing the platelet-activating factor (PAF) pathway, observably playing a role in platelet gathering resistance with C50 values similar to those of other marine PLs. PL components relevant to PC as well as PE ramifications obtained the highest anti-PAF activity (87). In conclusion, experiments indicated that marine PLs all had thrombotic resistance by protecting human platelets from the gathering induced by platelet agonists. Specifically, salmon heads, herring heads, herring filets, and minced boarfish could have anti-PAF activity, resembling the salmon PLs mentioned above. In addition, minced boarfish could obtain the highest collagen resistance activity with anti-PAF activity, while salmon heads had the highest anti-thrombin and anti-ADP activities. Herring head and herring filet PLs were demonstrated to have similar thrombotic resistance activity. Studies have suggested that each PL contains abundant omega-3 polyunsaturated fatty acids (ω3PUFAs), such as docosahexaenoic acid and eicosapentaenoic acid, at a ω3/ω6 ratio (88). Further investigations revealed that ω3PUFA could decrease TG. It was proven that the inhibition of non-esterified ω3PUFA transmission to the liver might be a fish oil (FO)-acting site because non-esterified ω3PUFAs are the most effective for VLDL-TG generation. FO could reduce intracellular steatolysis in adipose cells through anti-inflammation in adipose tissue while promoting extracellular steatolysis with the help of LDL in adipose tissue, heart tissue, and skeletal muscle and elevating β-oxidation in the liver and skeletal muscle so that the transmission of ω3PUFAs to the liver could be decreased. Additionally, FO could downregulate TG in plasma with the activation of transcription factors that affected metabolism (89).

Another study also revealed that FO could protect against arteriosclerosis by activating oxidase (90). As indicated by new experiments, FO treatment could promote endothelial progenitor cells and inhibit endothelial microparticles to defend the integrity of endotheliocytes (91). ω3PUFAs are believed to defend against arteriosclerosis as well as plaque disruption by inflammatory resistance (92). Emerging evidence indicates that ω3PUFAs can influence enteric microorganisms to protect the cardiovascular system by inhibiting the TMA produced by bacteria, promoting anti-inflammatory-active bacteria to create butyrate and downregulating proinflammatory fingers. In addition, ω3PUFAs defend gut barrier integrity to hold back intestinal contents from circulation (93). However, it was also reported that ω6PUFAs at high concentrations might reduce the influence of ω3PUFAs on the cardiovascular system (94). Another finding revealed that docosahexaenoic acid and eicosapentaenoic acid administration with 1-g values might help improve metabolic disturbance (95).

## Marine-Derived Angiotensin-Converting Enzyme Inhibitory Peptides

Previous studies indicated that the two angiotensin-converting enzyme (ACE) inhibitory peptides extracted from *G. lemaneiformis*, FQIN [M (O)] CILR and TGAPCR, could non-competitively suppress ACE. The anti-ACE activity of the two peptides mentioned above was demonstrated to be their link to ACE by hydrogen bonds. In animal tests, they were indicated to obtain hypotensive ability (96). Another peptide (97) that was isolated from *Cyclina sinensis* was indicated to have not only C-terminus- but also N-terminus-hydrophobic amino acid residues, contributing to its anti-ACE activity (98). It was also reported that C- and N-terminal peptide array differences might influence its anti-ACE activity (99).

## Challenges and Future Outlooks

Currently, CAD, which is an inflammatory disease based on atherosclerosis, is still believed to be the most prolegomena cause of death worldwide. It was reported that the actual therapy for CAD included anti-ischemic drugs, antiplatelet drugs, anticoagulant drugs, and lipid-lowering drugs (100). With the rapid development of medical treatment worldwide, drugs for CAD have been updated to a great degree. Angiotensin receptor-neprilysin inhibitors and sodium-glucose cotransporter 2 inhibitors have shown, in recent years, many gratifying results. However, there is no denying that CAD still has tremendous residual risks, which demands continued exploration for new drugs. Recently, marine natural products have been demonstrated to have potential activity for CAD treatment (101). The abundant activity of marine drugs includes platelet resistance, coagulant resistance, lipid-lowering effects, inflammation resistance, and oxidation resistance; pivotally, they have few untoward effects and should never be overlooked. We should place emphasis on this field, which has received little attention previously. Therefore, in this article, we chiefly summarized four varieties of marine natural products, including organic small molecules, polysaccharides, proteins and bioactive peptides, and lipids, which were indicated to be helpful for CAD (Table 1).

**Table 1:** Summary of marine natural products in coronary artery disease.

**Classification**	**Compounds**	**Signaling pathways**	**Function**	**References**
Organic small molecules	Fucoxanthin	PPARα, PGC1α, PPARγ, UCP-1, GLUT4, GCK, GSY, p-ACC, CPT-1, IRS-1, PI3K, AKT, AMPK	Promote	(8–11)
		ACC, IL-6, PEPCK, GSK3β, FAS	Suppress	(8, 11)
	Saponins	SIRT1	Promote	(14)
	Astaxanthin	SREBP-2, PPARα	Promote	(19)
		VeGF–VeGFr2–FaK	Suppress	(21)
	Xyloketal B	CPT1A, Bcl-2, NO	Promote	(27)
		SREBP-1c	Suppress	(27)
	DSW	LDLR, SREBP-1α, SREBP-2	Promote	(6)
		PCSK9 mRNA, AMPK, PPARα, CPT-1, ACO, HMGCR, PCSK9	Suppress	(6)
	Terpenes	PI3K	Suppress	(33)
	Benzoic acid derivative	ATF-3	Promote	(34)
		JNK, ERK, activator protein-1, NF-κB	Suppress	(79)
	Sponge extract	PPRE, SR-B1	Promote	(12)
	Asperlin	IL-10, IL-4	Promote	(37)
		iNOS, IL-1β, TNFα, MCP-1, IL-6	Suppress	(12, 37)
Polysaccharides	Fucoidan	HIF-1/VEGF, miR-29b-DNMT3B-MTSS1-mediated TGF-signaling, HIF-1/VEGF-C, PI3K/Akt/mTOR pathway, JNK/c-Jun/AP-1 pathway, PCSK9, SREBPs	Suppress	(41, 43)
		PPARα, LXRβ/ABC, SR-B1, LDLR, CYP7A1, NPC1L1, ABCG5, ABCG8	Promote	(41)
	Alginate	SREBP-2	Promote	(46)
		PCSK9	Suppress	(46)
	Ulvan	IL-1β, IL-6	Suppress	(49)
	Chitosan	CYP7A1, GLP-1, Nrf2/ARE, JNK MAPK, LDL-R, SR-BI, ABCAI	Promote	(54, 56, 57)
		CD36, PXR, DGAT2, LXRα, HMGCR, SMYD3, PPARγ	Suppress	(52, 54)
	Enteromorpha prolifera polysaccharides	HIF-1α	Promote	(60)
	Chondroitin sulfate	VCAM-1, ICAM 1, ephrin-B2, CD36, CD146	Suppress	(76)
Proteins and bioactive peptides	Proteins and bioactive peptides	Pon2	Suppress	(78)
Lipids	Lipids	PAF	Suppress	(87)

Experiments and investigations have indicated several marine natural products that are effective for CAD. Here, we particularly discussed the functions and mechanisms of these active substances on CAD, including antiplatelet, anticoagulant, lipid-lowering, anti-inflammatory, and antioxidant activities. According to current knowledge, the mechanism of CAD is complex. Interestingly, we focused mainly on the active substances extracted from marine natural products and indicated that they had something to do with CAD through their lipid-lowering, thrombus resistance, and inflammation resistance functions. A large number of *in vivo* and *in vitro* experiments could provide evidence for these results. For the drugs mentioned above, the molecular mechanisms of fucoxanthin, saponins, astaxanthin, xyloketal B, DSW, terpenes, benzoic acid derivative, sponge extract, asperlin, fucoidan, alginate, ulvan, chitosan, enteromorpha prolifera polysaccharides, chondroitin sulfate, proteins, and bioactive peptides as well as lipids have been clearly elucidated. Nevertheless, the functions of treating CAD with the other drugs were confirmed only by biochemical indices, the mechanisms behind, which remained to be further discussed in the case of lacking high-quality clinical evidence. There is much room for later scholars to study the signaling pathways behind carrageenan, PPs, and ACE inhibitory peptides. Furthermore, all studies rested on the active substances and paid less attention to developing marine drugs that could help treat CAD. As a consequence, the studies could not guide clinical medication directly. There is still a long way to go to employ marine drugs for the treatment of CAD. Further investigations could contribute to this field.

## Author Contributions

BL and NG designed the study. BL, X-YC, and NG acquired and researched data for the article and discussed its content. BL and X-YC wrote the manuscript. NG revised the manuscript. All authors read and approved the final manuscript.

## Funding

This work was partly supported by the National Natural Science Foundation of China (81774229), Research and Practice Innovation Plan for Postgraduates of Jiangsu, China (KYCX21_1641), Jiangsu Universities Nursing Advantage Discipline Project (2019YSHL095), and Jiangsu Leading Talent Project of Traditional Chinese Medicine (Jiangsu TCM 2018 No. 4).

## Conflict of Interest

The authors declare that the research was conducted in the absence of any commercial or financial relationships that could be construed as a potential conflict of interest.

## Publisher's Note

All claims expressed in this article are solely those of the authors and do not necessarily represent those of their affiliated organizations, or those of the publisher, the editors and the reviewers. Any product that may be evaluated in this article, or claim that may be made by its manufacturer, is not guaranteed or endorsed by the publisher.
